# TiGeS_3_

**DOI:** 10.1107/S1600536812037087

**Published:** 2012-09-01

**Authors:** Pilsoo Kim, Hoseop Yun

**Affiliations:** a Division of Energy Systems Research and Department of Chemistry, Ajou University, Suwon 443-749, Republic of Korea

## Abstract

The new ternary titanium(II) thio­germanate(IV), TiGeS_3_, was synthesized using the reactive halide flux method. The title compound shows features of a ribbon-type structure formed from double chains composed of edge-sharing octa­hedral TiS_6_ and pyramidal GeS_3_ units, with all atoms in the asymmmetric unit positioned on mirror planes. While the TiS_6_ octa­hedron is regular, the coordination around the Ge atom is rather irregular, which can be described as [3 + 3]. Three S atoms build up a triangle that is bound to the Ge atom, the coordination of which is augmented by three additional S atoms at considerably longer distances. The charge balance can formally be described as [Ti^4+^][Ge^2+^][S^2−^]_3_.

## Related literature

For related structures, see: Brasseur & Pauling (1938 ▶) for NH_4_CdCl_3_; Kniep *et al.* (1982 ▶) for Sn^II^Sn^IV^S_3_. For isostructural compounds, see: Lelieveld & Ijdo (1978 ▶) for PbZrS_3_; Gressier *et al.* (1987 ▶) for Sn_1.2_Ti_0.8_S_3._ For usual bond lengths and angles for the TiS_6_ octa­hedron, see: Jandali *et al.* (1980 ▶) for TiP_2_S_6_. A similar coordination of Ge to that observed in the title compound can be found in divalent germanium sulfide, GeS, see: Bissert & Hesse (1978 ▶).

## Experimental

### 

#### Crystal data

TiGeS_3_
*M* *_r_* = 216.68
Orthorhombic, 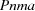

*a* = 8.9263 (8) Å
*b* = 3.4673 (3) Å
*c* = 13.2596 (15) Å
*V* = 410.39 (7) Å^3^
*Z* = 4
Mo *K*α radiation
μ = 10.56 mm^−1^
*T* = 290 K
0.60 × 0.08 × 0.06 mm

#### Data collection

Rigaku R-AXIS RAPID diffractometer
Absorption correction: multi-scan (*ABSCOR*; Higashi, 1995 ▶) *T*_min_ = 0.674, *T*_max_ = 1.000
3802 measured reflections
550 independent reflections
525 reflections with *I* > 2σ(*I*)
*R*_int_ = 0.031

#### Refinement

*R*[*F* ^2^ > 2σ(*F* ^2^)] = 0.032
*wR*(*F* ^2^) = 0.076
*S* = 1.16
550 reflections
32 parameters
Δρ_max_ = 1.69 e Å^−3^
Δρ_min_ = −1.44 e Å^−3^

### 

Data collection: *RAPID-AUTO* (Rigaku, 2006 ▶); cell refinement: *RAPID-AUTO*; data reduction: *RAPID-AUTO*; program(s) used to solve structure: *SHELXS97* (Sheldrick, 2008 ▶); program(s) used to refine structure: *SHELXL97* (Sheldrick, 2008 ▶); molecular graphics: *DIAMOND* (Brandenburg, 1999 ▶); software used to prepare material for publication: *WinGX* (Farrugia, 1999 ▶).

## Supplementary Material

Crystal structure: contains datablock(s) global, I. DOI: 10.1107/S1600536812037087/ru2041sup1.cif

Structure factors: contains datablock(s) I. DOI: 10.1107/S1600536812037087/ru2041Isup2.hkl

Additional supplementary materials:  crystallographic information; 3D view; checkCIF report

## Figures and Tables

**Table 1:** Selected bond lengths (Å)

Ti—S1^i^	2.4238 (12)
Ti—S2	2.4246 (12)
Ti—S3^ii^	2.4286 (8)
Ti—S3	2.4286 (8)
Ti—S1	2.4469 (8)
Ti—S1^ii^	2.4469 (8)
Ge—S2^iii^	2.3970 (8)
Ge—S2	2.3970 (8)
Ge—S3	2.4754 (11)
Ge—S1^iv^	3.0558 (11)
Ge—S3^v^	3.4360 (10)
Ge—S3^vi^	3.4360 (10)

Symmetry codes: (i) 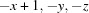; (ii) 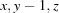; (iii) 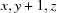; (iv) 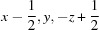; (v) 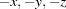; (vi) 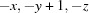.

**Fig. 1.**
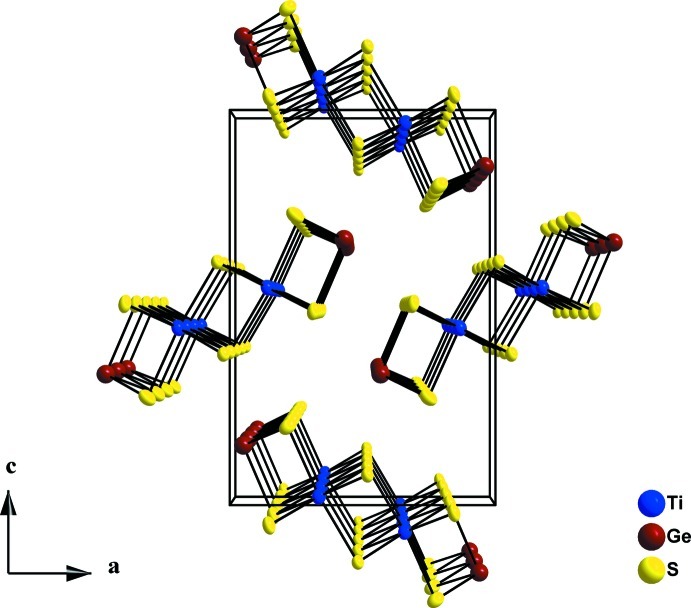
A perspective view of TiGeS3 down the b axis showing the ribbon-type structure of the chain.

**Fig. 2.**
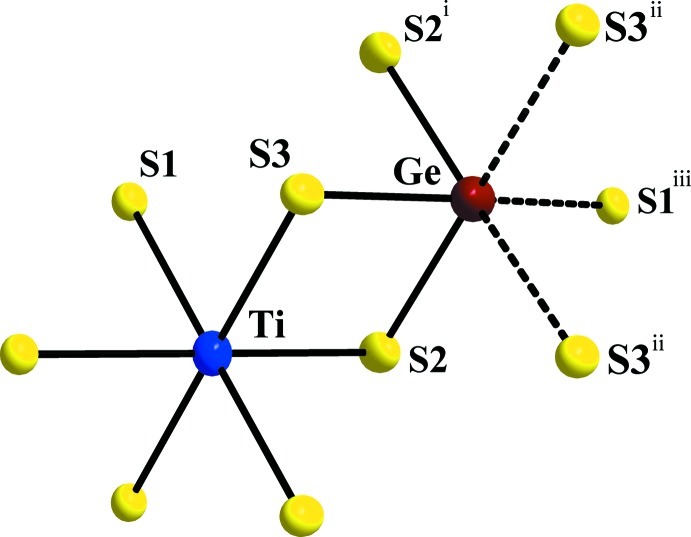
A view showing the local coordination environments of Ti and Ge atoms with the atom labelling scheme. Displacement ellipsoids are drawn at the 80% probability level. [Symmetry codes: (i) x, 1+y, z; (ii) -x, 1/2+y, -z; (iii) -1/2+x, 1/2-y, 1/2-z.]

## supplementary crystallographic information

### Comment

During an effort to find a new phase in the A—Ti—Ge—S system (A=alkali metals), a new compound was isolated. Here we report the synthesis and structure of the new ternary thiogermanate, TiGeS_3_.

The title compound shows the features of the ribbon-type structure (Fig. 1). Its structure is closely related to that of NH_4_CdCl_3_ (Brasseur & Pauling, 1938) and it is isostructural with the previously reported Sn^II^Sn^IV^S_3_ (Kniep *et al.*, 1982), PbZrS_3_ (Lelieveld & Ijdo, 1978), and Sn_1.2_Ti_0.8_S_3_ (Gressier *et al.*, 1987). The Ti atom is coordinated by six sulfur atoms in an octahedral arrangement (Fig. 2). The TiS_6_ octahedra share an edge to form the one-dimensional chains along the *b* axis. These chains are fused together sharing two S atoms to form the double chain. These double octahedral chains are capped by the Ge atom to complete the one-dimensional chain.

While the TiS_6_ octahedra are regular and the Ti—S distances are in good agreement with those found in other titanium sulfides (Jandali *et al.*, 1980), the coordination around the Ge atom is rather irregular. It can be described as [3 + 3]. Three S atoms built up a triangle that is bound to the Ge atom (Ge—S, 2.397 (1)–2.475 (1) Å; S—Ge—S, 91.13 (3)–92.65 (4) °), the coordination of which is augmented by three additional S atoms at considerably longer distances of 3.056 (1)–3.436 (1) Å. The similar Ge coordination observed in the title compound can be found in divalent germanium sulfide, GeS (Bissert & Hesse, 1978) and the charge balance can be described by [Ti^4+^][Ge^2+^][S^2-^]_3_.

### Experimental

The title compound, TiGeS_3_ was prepared by the reaction of elements with the use of the reactive halide-flux technique. A combination of the pure elements, Ti powder(Aldrich 99.7%), Ge powder(CERAC 99.9%) and S powder(Aldrich 99.999%) were mixed in a fused silica tube in molar ratio of Ti:Ge:*S*=4:2:7 and then KCl(CERAC 99.9%) was added. The mass ratio of the reactants and the halide was 1:2. The tube was evacuated to 0.133 Pa, sealed, and heated gradually (20 K/h) to 923 K, where it was kept for 72 h. The tube was cooled to room temperature at the rate 3 K/h. The excess halide was removed with distilled water and black needle-shaped crystals were obtained. The crystals are stable in air and water. A qualitative X-ray fluorescence analysis of the needles indicated the presence of Ti, Ge, and S. The composition of the compound was determined by single-crystal X-ray diffraction.

### Refinement

The Ti site in Sn_1.2_Ti_0.8_S_3_ has been reported to be occupied by disordered Ti^4+^ and Sn^4+^ ions (Gressier *et al.*, 1987) and the nonstoichiometry of the Ti site in the title compound was checked by refining the occupancy and anisotropic displacement parameters while those of the other atoms were fixed. With the nonstoichiometric model, both parameters were not changed significantly and the residuals (wR2, R1 indices) remained the same. The highest peak(1.69 e/Å-3) and the deepest hole (-1.44 e/ Å-3) are 0.77 Å and 0.86 Å from the atom Ge, respectively.

### Crystal data

**Table:**

TiGeS_3_	*F*(000) = 408
*M**_r_* = 216.68	*D*_x_ = 3.507 Mg m^−^^3^
Orthorhombic, *P**n**m**a*	Mo *K*α radiation, λ = 0.71073 Å
Hall symbol: -P 2ac 2n	Cell parameters from 3487 reflections
*a* = 8.9263 (8) Å	θ = 3.1–27.4°
*b* = 3.4673 (3) Å	µ = 10.56 mm^−^^1^
*c* = 13.2596 (15) Å	*T* = 290 K
*V* = 410.39 (7) Å^3^	Needle, black
*Z* = 4	0.60 × 0.08 × 0.06 mm

### Data collection

**Table:**

Rigaku R-AXIS RAPID diffractometer	525 reflections with *I* > 2σ(*I*)
Graphite monochromator	*R*_int_ = 0.031
ω scans	θ_max_ = 27.4°, θ_min_ = 3.1°
Absorption correction: multi-scan (*ABSCOR*; Higashi, 1995)	*h* = −11→11
*T*_min_ = 0.674, *T*_max_ = 1.000	*k* = −4→4
3802 measured reflections	*l* = −17→17
550 independent reflections	

### Refinement

**Table:**

Refinement on *F*^2^	Primary atom site location: structure-invariant direct methods
Least-squares matrix: full	Secondary atom site location: difference Fourier map
*R*[*F*^2^ > 2σ(*F*^2^)] = 0.032	*w* = 1/[σ^2^(*F*_o_^2^) + (0.0422*P*)^2^ + 0.885*P*] where *P* = (*F*_o_^2^ + 2*F*_c_^2^)/3
*wR*(*F*^2^) = 0.076	(Δ/σ)_max_ < 0.001
*S* = 1.16	Δρ_max_ = 1.69 e Å^−^^3^
550 reflections	Δρ_min_ = −1.44 e Å^−^^3^
32 parameters	Extinction correction: *SHELXL97* (Sheldrick, 2008)
0 restraints	Extinction coefficient: 0.049 (3)

### Special details

**Table:**

Geometry. All e.s.d.'s (except the e.s.d. in the dihedral angle between two l.s. planes) are estimated using the full covariance matrix. The cell e.s.d.'s are taken into account individually in the estimation of e.s.d.'s in distances, angles and torsion angles; correlations between e.s.d.'s in cell parameters are only used when they are defined by crystal symmetry. An approximate (isotropic) treatment of cell e.s.d.'s is used for estimating e.s.d.'s involving l.s. planes.
Refinement. Refinement of *F*^2^ against ALL reflections. The weighted *R*-factor *wR* and goodness of fit *S* are based on *F*^2^, conventional *R*-factors *R* are based on *F*, with *F* set to zero for negative *F*^2^. The threshold expression of *F*^2^ > σ(*F*^2^) is used only for calculating *R*-factors(gt) *etc*. and is not relevant to the choice of reflections for refinement. *R*-factors based on *F*^2^ are statistically about twice as large as those based on *F*, and *R*- factors based on ALL data will be even larger.

### Fractional atomic coordinates and isotropic or equivalent isotropic displacement parameters (Å^2^)

**Table:**

	*x*	*y*	*z*	*U*_iso_*/*U*_eq_	
Ti	0.34548 (8)	−0.25	0.04861 (5)	0.0133 (3)	
Ge	0.06572 (5)	0.25	0.16292 (4)	0.0227 (2)	
S1	0.51690 (10)	0.25	0.10897 (7)	0.0102 (3)	
S2	0.23401 (12)	−0.25	0.21536 (7)	0.0146 (3)	
S3	0.17500 (11)	0.25	−0.00866 (8)	0.0125 (3)	

### Atomic displacement parameters (Å^2^)

**Table:**

	*U*^11^	*U*^22^	*U*^33^	*U*^12^	*U*^13^	*U*^23^
Ti	0.0111 (4)	0.0142 (4)	0.0145 (4)	0	0.0012 (3)	0
Ge	0.0145 (3)	0.0144 (3)	0.0393 (4)	0	0.00642 (19)	0
S1	0.0083 (5)	0.0108 (5)	0.0116 (5)	0	−0.0002 (3)	0
S2	0.0167 (5)	0.0124 (5)	0.0148 (5)	0	0.0045 (4)	0
S3	0.0089 (5)	0.0111 (5)	0.0174 (5)	0	−0.0021 (3)	0

### Geometric parameters (Å, º)

**Table:**

Ti—S1^i^	2.4238 (12)	Ge—S3	2.4754 (11)
Ti—S2	2.4246 (12)	Ge—S1^iv^	3.0558 (11)
Ti—S3^ii^	2.4286 (8)	Ge—S3^v^	3.4360 (10)
Ti—S3	2.4286 (8)	Ge—S3^vi^	3.4360 (10)
Ti—S1	2.4469 (8)	S1—Ti^i^	2.4238 (12)
Ti—S1^ii^	2.4469 (8)	S1—Ti^iii^	2.4469 (8)
Ge—S2^iii^	2.3970 (8)	S2—Ge^ii^	2.3970 (8)
Ge—S2	2.3970 (8)	S3—Ti^iii^	2.4286 (8)
			
S1^i^—Ti—S2	173.78 (5)	S1—Ti—S1^ii^	90.22 (4)
S1^i^—Ti—S3^ii^	92.75 (4)	S2^iii^—Ge—S2	92.65 (4)
S2—Ti—S3^ii^	91.60 (4)	S2^iii^—Ge—S3	91.13 (3)
S1^i^—Ti—S3	92.75 (4)	S2—Ge—S3	91.13 (3)
S2—Ti—S3	91.60 (4)	Ti^i^—S1—Ti	92.00 (3)
S3^ii^—Ti—S3	91.10 (4)	Ti^i^—S1—Ti^iii^	92.00 (3)
S1^i^—Ti—S1	88.00 (3)	Ti—S1—Ti^iii^	90.22 (4)
S2—Ti—S1	87.61 (3)	Ge^ii^—S2—Ge	92.65 (4)
S3^ii^—Ti—S1	179.11 (4)	Ge^ii^—S2—Ti	89.58 (3)
S3—Ti—S1	89.33 (2)	Ge—S2—Ti	89.58 (3)
S1^i^—Ti—S1^ii^	88.00 (3)	Ti^iii^—S3—Ti	91.10 (4)
S2—Ti—S1^ii^	87.61 (3)	Ti^iii^—S3—Ge	87.68 (3)
S3^ii^—Ti—S1^ii^	89.33 (2)	Ti—S3—Ge	87.68 (3)
S3—Ti—S1^ii^	179.11 (4)		

Symmetry codes: (i) −*x*+1, −*y*, −*z*; (ii) *x*, *y*−1, *z*; (iii) *x*, *y*+1, *z*; (iv) *x*−1/2, *y*, −*z*+1/2; (v) −*x*, −*y*, −*z*; (vi) −*x*, −*y*+1, −*z*.
